# Curdlan (*Alcaligenes faecalis*) (1→3)-β-d-Glucan Oligosaccharides Drive M1 Phenotype Polarization in Murine Bone Marrow-Derived Macrophages via Activation of MAPKs and NF-κB Pathways

**DOI:** 10.3390/molecules24234251

**Published:** 2019-11-22

**Authors:** Jun Liu, Jiqing Tang, Xiuting Li, Qiaojuan Yan, Junwen Ma, Zhengqiang Jiang

**Affiliations:** 1 Beijing Advanced Innovation Center for Food Nutrition and Human Health, College of Food Science and Nutritional Engineering, China Agricultural University, Beijing 100083, China; junliu@cau.edu.cn (J.L.); b20143060289@cau.edu.cn (J.T.); mjw@cau.edu.cn (J.M.); 2 Beijing Advanced Innovation Center for Food Nutrition and Human Health, Beijing Technology and Business University, Beijing 100048, China; lixt@btbu.edu.cn; 3 Key Laboratory of Food Bioengineering (China National Light Industry), College of Food Science and Nutritional Engineering, China Agricultural University, No.17 Qinghua Donglu, Haidian District, Beijing 100083, China; 4 Bioresource Utilization Laboratory, College of Engineering, China Agricultural University, Beijing 100083, China; yanqj@cau.edu.cn

**Keywords:** curdlan (1→3)-β-d-glucan oligosaccharides, macrophage polarization, mitogen-activated protein kinases, nuclear factor-kappa B, receptor blocking

## Abstract

Functional oligosaccharides, particularly curdlan (1→3)-β-d-glucan oligosaccharides (GOS), play important roles in modulating host immune responses. However, the molecular mechanisms underlying the immunostimulatory effects of GOS on macrophage polarization are not clear. In this work, GOS (5–1000 µg/mL) were non-toxic to bone marrow-derived macrophages (BMDMs) with improved pinocytic and bactericidal capacities. Incubation with GOS (100 µg/mL) induced M1 phenotype polarization of BMDMs as evidenced by increased CD11c^+^/CD86^+^ (10.1%) and M1 gene expression of inducible nitric oxide synthase, interleukin (IL)-1β, and chemokine C-C-motif ligand 2. Accordingly, the secretion of cytokines IL-1β, IL-6, monocyte chemotactic protein-1, and tumor necrosis factor-α, as well as the nitrite release of BMDMs were increased by GOS (100 µg/mL). Expression of mitogen-activated protein kinases (MAPKs) of phosphorylated (p)-c-Jun amino-terminal kinase, p-extracellular signal regulated kinase, and p-p38 in BMDMs were increased by GOS, as well as the p-Stat1. Moreover, nuclear factor-kappa B (NF-κB) p-p65 expression in BMDMs was promoted by GOS while it suppressed IκBα expression. Receptor blocking with anti-CR3 (CD11b/CD18) and anti-toll-like receptor (TLR) 2 antibodies diminished GOS induced M1 phenotype polarization with reduced mRNA expression of M1 genes, decreased cytokine and nitrite releases, and suppressed signaling pathway activation. Thus, CR3 (CD11b/CD18) and TLR2 mediated activation of MAPKs and NF-κB pathways are responsible for GOS induced polarization of BMDMs.

## 1. Introduction

Macrophages are innate immune cells that present in tissues with functional diversities [1]. They are heterogeneous and their remarkable plasticity and versatility are their hallmarks [2,3]. In response to surrounding microenvironmental stimuli and signaling molecules, macrophages can differentiate into different phenotypes, which is commonly termed as “macrophage polarization” [4]. The naïve macrophages (Mϕ) are readily phenotypically polarized into two almost exactly oppositely functioned subsets: classically activated macrophages (M1) or alternatively activated macrophages (M2) [1]. M1 macrophages can be activated either by toll-like receptor (TLR) ligands of lipopolysaccharide (LPS) or cytokines of interferon-γ (IFN-γ), and granulocyte macrophage colony stimulating factor (GM-CSF) [5]. They have high antigen presentation capacity and high production of pro-inflammatory cytokines and mediators of tumor necrosis factor (TNF)-α, interleukin (IL)-6, IL-1α, IL-1β, IL-12, IL-23, nitric oxide (NO), and chemokine (C-X-C motif) ligand 9 (CXCL9), but low levels of IL-10 [6,7]. M1 macrophages can activate the nicotinamide adenine dinucleotide phosphate (NADPH) oxidase system and induce the production of reactive oxygen species (ROS) [8]. Those facilitate the participation of M1 macrophages in the removal of microbial infections and tumor cells, mediation of tissue damage induced by ROS, impairment of wound healing, and tissue regeneration [8,9]. M2 macrophages can be polarized by cytokines IL-4, IL-10, IL-13, IL-21, and IL-33, glucocorticoids, immune complexes (IC), and LPS [5]. Based on the applied stimuli and the resultant transcriptional profiles, they can be further divided into subcategories of M2a, M2b, M2c, and M2d [9]. M2 macrophages are characterized by the high production of both IL-10 and tumor growth factor (TGF)-β, but low levels of IL-12 [6,7]. They are involved in inflammation resolution, Th2 responses and parasite clearance, tissue remodeling and wound healing, angiogenesis, immune tolerance, tumor formation, and progression [8,9]. Thus, a balance between macrophage M1 and M2 phenotypes is generally thought to be critical for the host defense mediation. Polarization of Mϕ into M1/M2 macrophages regulation may serve as a promising therapeutic strategy for treatment of inflammation-related diseases, such as metabolic syndromes, obesity, cancer, asthma, allergic disorders, autoimmune diseases, and atherosclerosis [10,11]. Extreme M1/M2 polarization has been widely utilized for mechanistic studies in vitro even though macrophages are a “spectrum” of populations with a continuum of functional phenotypes under physiological conditions [1].

Polysaccharides from sources of *Platycladus orientalis* (L.) Franco leaves [11], *Echinacea purpurea* [12], edible mushrooms (*Pleurotus citrinopileatus*) [13], *Cordyceps militaris* [14], maca (*Lepidium meyenii*) [15], Astragali Radix decoction (*Astragalus*) [16], and *Strongylocentrotus nudus* eggs [17] have been found to regulate macrophage polarization. Natural product β-d-glucans, particularly (1→3)-β-d-glucans derived from yeast, fungi, bacteria, or barley, also show immunomodulatory effects and display multiple pharmacological functions through immune regulation [18,19,20,21]. They are recognized by the innate immune system, which plays important roles in host defense through leukocyte activation and the production of inflammatory mediators [18]. Receptors of complement receptor 3 (CR3), TLRs, and Dectin-1 that express on immune cell surface translate the recognition of β-d-glucans into intracellular signaling and thus immune responses [19]. Interestingly, yeast derived particulate (1→3)-β-d-glucan showed immunostimulating activity with potential therapeutic efficacy in tumor-bearing mice. It induced the conversion of M1 polarized alternatively activated macrophages or immunosuppressive tumor-associated macrophages to M2 phenotype through the dectin-1-dependent canonical spleen tyrosine kinase (Syk)–Card9–Erk pathway [20]. However, the binding affinity between Dectin-1 and laminarin, a low molecular weight (1→3)-β-d -glucan with (1 6)-β side chains, is dependent on the physicochemical properties, purity, and structure features [21]. Curdlan is a microbial extracellular homo-polysaccharide of (1→3)-β-d-glucan which has been approved by the U.S. Food and Drug Administration for utilization in the food industry [22]. Due to its remarkable rheological and gelation properties, curdlan has been widely applied as a food stabilizer, thickener, texturizer, and/or formation or processing aid [23,24,25]. Curdlan shows pleiotropic immunostimulatory effects through the innate immune response activation [26,27]. Those were considered to be associated with the improved anti-coagulant, anti-bacterial, anti-fungal, anti-viral, anti-tumor, and wound repair activities of curdlan [5,19,28]. However, the water insolubility and unique gelation property of curdlan affect its biological performance, and subsequently its potential applications in the food industry [27,28,29,30].

Curdlan (1→3)-β-d-glucan oligosaccharides (GOS) prepared through chemical or enzymatic hydrolysis has shorter chain length [30]. GOS with a degree of polymerization (DP) of 2−4 (*Alcaligenes faecalis* var. *myxogenes*) induced cytokines production of splenocytes and bone marrow-derived dendritic cells [31]. As compared with curdlan, GOS (DP 2−5) prepared by enzymatic hydrolysis using GH family 64 β-(1→3)-glucanase (*Rm*Lam81A) showed superior immunostimulatory effects in cyclophosphamide induced immunosuppressed mice [32]. Similar improvement on immunostimulatory effects have been reported on paramylon derived (1→3)-β-d-glucan oligosaccharides using exo-β-1, 3-glucanase. (1→3)-β-d-glucan oligosaccharides with shorter chain length (DP 2−7) led to higher level tumor necrosis factor (TNF)-α production than those of longer chain lengths (DP 2−59 and DP 2−38) [33]. However, the effects of GOS on macrophage polarization and the underlying molecular mechanisms have not been studied yet.

In this study, bone marrow-derived macrophages (BMDMs) were isolated and differentiated from healthy male C57BL/6J mice. Effects of GOS on cytotoxicity, pinocytic capacity, bactericidal function, and polarization of BMDMs were investigated. The signaling pathways and receptors responsible for GOS recognition and immune response activation were explored to elucidate the underlying molecular mechanisms.

## 2. Results

### 2.1. Proliferation, Pinocytosis, and Bactericidal Function of BMDMs

**Figure 1:**
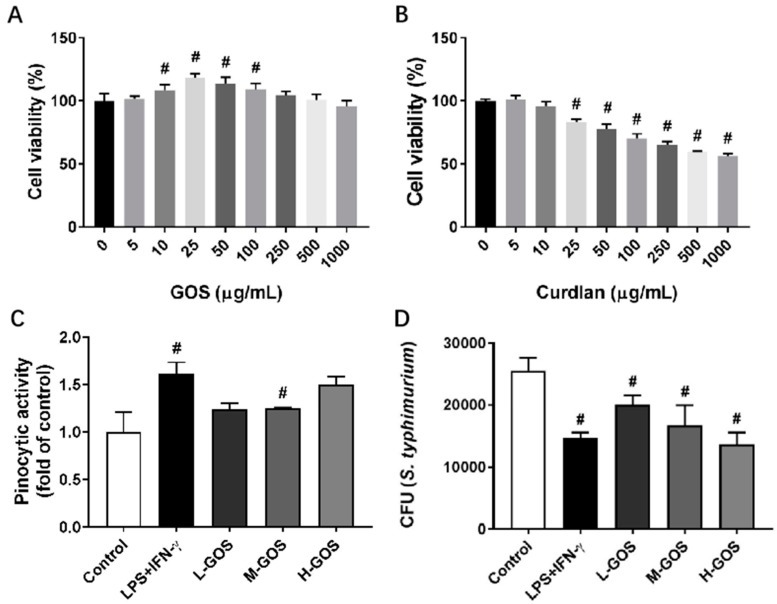
Cytotoxicity of (1→3)-β-d-glucan oligosaccharides (GOS) and the effects on pinocytic capacity and bactericidal function of bone marrow-derived macrophages (BMDMs). Cell viability of BMDMs was measured by MTT (3-(4,5-dimethylthiazol-2-yl)-2,5-diphenyltetrazolium bromide) assay after incubation with GOS (5–1000 μg/mL, (**A**)) and curdlan (5–1000 μg/mL, (**B**)) for 24 h. Pinocytosis of BMDMs was measured as the cellular uptake of fluorescein isothiocyanate (FITC)-dextran using flow cytometry after incubation with GOS (25−100 μg/mL) for 12 h at 37 °C (**C**). Bactericidal function of BMDMs was analyzed by counting the colony forming unit (CFU) of intracellular *S. typhimurium* after incubation with GOS (25−100 μg/mL) for 24 h (**D**). **^#^**—Indicated significant difference versus the control group at *p* < 0.05.

Endotoxin contaminations in GOS (5–1000 µg/mL) were analyzed with a Pierce LAL Chromogenic Endotoxin Quantitation kit (Thermo Scientific, Rockford, IL, USA) following the manufacturer’s instructions. No detectable endotoxin contaminations were identified in GOS (data not shown). The cytotoxicity of GOS and curdlan on BMDMs were evaluated Figure 1A,B. GOS were non-cytotoxic against BMDMs after 48 h incubation within the concentration range of 5–1000 μg/mL. At a concentration of 10–100 μg/mL, GOS showed proliferative effects on BMDMs (Figure 1A). However, cell viability of BMDMs was significantly decreased after treatment with curdlan at concentrations higher than 25 μg/mL, which indicated the cytotoxicity of curdlan on BMDMs.

Effects of GOS on pinocytic capacity of BMDMs were analyzed through the uptaking of fluorescein isothiocyanate (FITC)-dextran (Figure 1C). As compared with the control, pretreatment with LPS (1 µg/mL) + INF-γ (100 ng/mL) significantly increased the uptake of FITC-dextran in BMDMs. GOS of 25 µg/mL (L-GOS) and 50 µg/mL (M-GOS) did not significantly change the pinocytic capacity of BMDMs. However, BMDMs treated with 100 µg/mL GOS (H-GOS) showed significantly increased FITC-dextran uptaking, which indicated the improved intracellular pathogen killing capacity of BMDMs. GOS also increased the bactericidal function of BMDMs as shown in Figure 1D. After LPS (1 µg/mL) + INF-γ (100 ng/mL) treatment, the colony-forming unit (CFU) of intracellular *Salmonella typhimurium* (*S. typhimurium*) was significantly decreased as compared with that of the control. BMDMs incubated with GOS showed decreased CFU of intracellular *S. typhimurium* in a dose-dependent manner, which confirmed the improved bacterial killing capacity of BMDMs by GOS.

### 2.2. GOS Promoted M1 Phenotype Polarization of BMDMs

**Figure 2:**
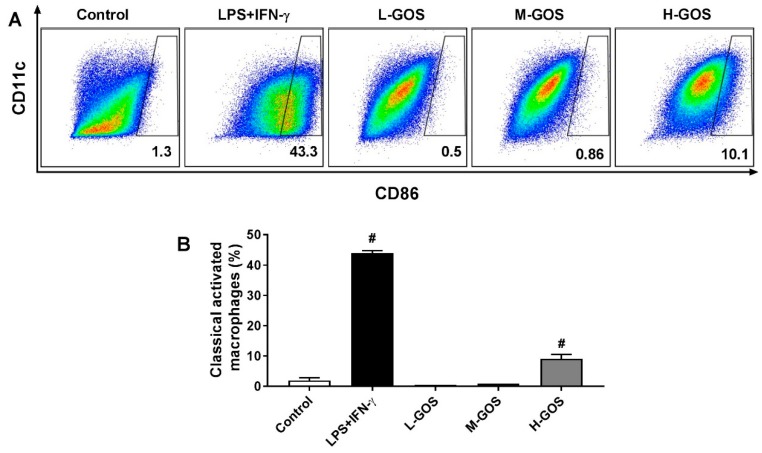
Effects of GOS on polarization of BMDMs. Expression of CD11c and CD86 on BMDMs cell surface (**A**) and accordingly the percentage of M1 phenotype macrophages (**B**) after GOS (25−100 μg/mL) treatment was analyzed by flow cytometry. **^#^**—Indicated significant difference versus the control group at *p* < 0.05.

Effects of GOS on the polarization of BMDMs isolated from C57BL/6 mice were examined by measuring the surface expression of CD11c and CD86. As shown in Figure 2, the percentage of CD11c^+^/CD86^+^ macrophages was significantly increased by the treatment of LPS (1 µg/mL) + INF-γ (100 ng/mL) (43.3%, *p* < 0.05) as compared with that of the control group (1.3%). At a dose lower than 50 μg/mL, L-GOS and M-GOS did not significantly change the percentage of CD11c^+^/CD86^+^ macrophages (0.5% and 0.86%, respectively). GOS at 100 μg/mL (H-GOS) significantly increased the percentage of CD11c^+^/CD86^+^ macrophages in the cultured BMDMs (10.1%, *p* < 0.05). Results indicated that GOS was able to induce M1 polarization of BMDMs in vitro.

### 2.3. GOS Induced Marker Expression on BMDMs’ Surface

**Figure 3:**
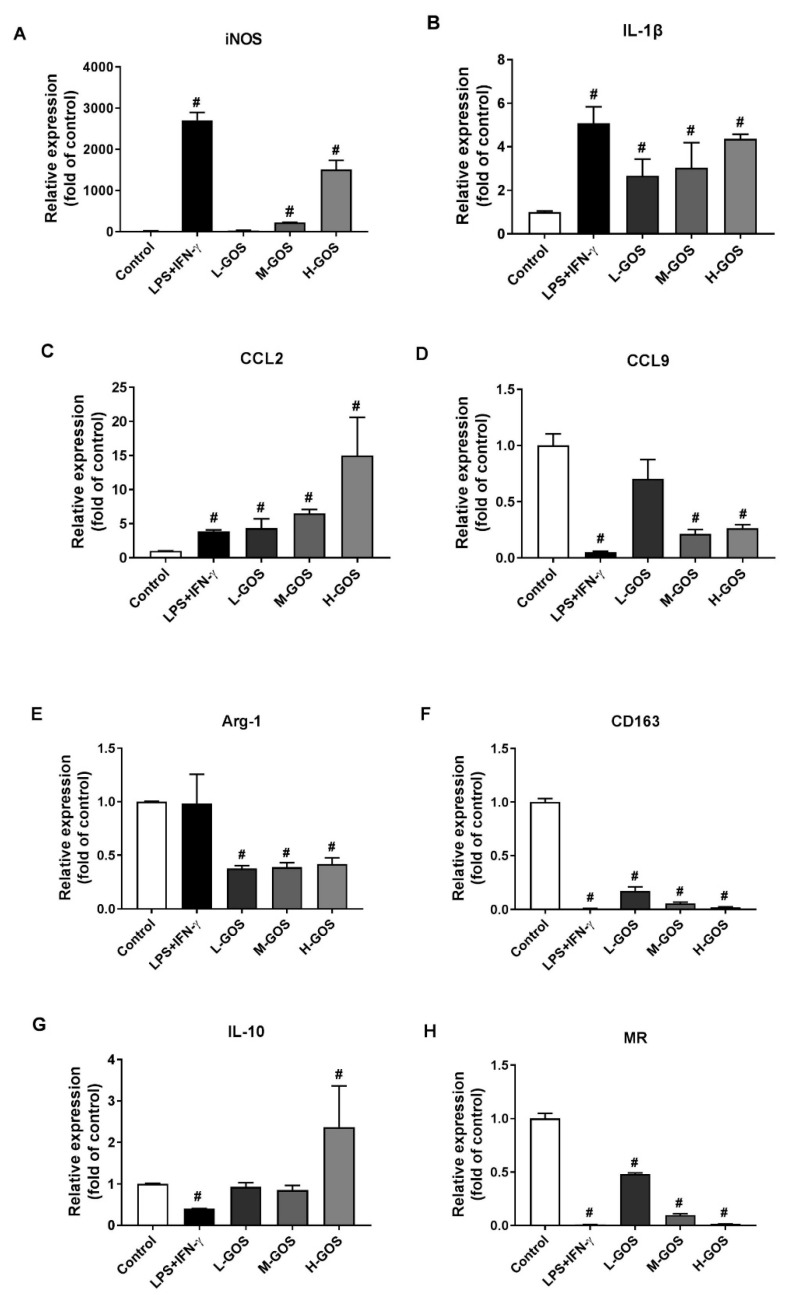
Effects of GOS on mRNA expression of M1 and M2 specific markers in BMDMs. The mRNA expression of M1 genes iNOS (**A**), IL-1β (**B**), CCL2 (**C**), CCL9 (**D**), as well as M2 genes Arg-1 (**E**), CD163 (**F**), IL-10 (**G**), and MR (**H**) in BMDMs were measured by real time-quantitative polymerase chain reaction (RT-qPCR) after GOS (25−100 μg/mL) treatment. **^#^**—Indicated significant difference versus the control group at *p* < 0.05.

To confirm the M1 phenotype polarization effects of GOS on BMDMs, mRNA expression of M1 genes inducible nitric oxide synthase (iNOS), IL-1β, and chemokine C-C-motif ligand (CCL)-2, and CCL9, and M2 genes Arg-1, CD163, IL-10, and mannose receptor (MR) were measured (Figure 3). As compared with the untreated cells (blank control), LPS (1 µg/mL) + INF-γ (100 ng/mL) significantly increased the expression of M1 genes iNOS (2704.1 folds), IL-1β (5.1 folds), and CCL2 (3.9 folds), while the CCL-9 (0.05 folds) expression was significantly suppressed (*p* < 0.05). The M2 gene expression of CD163 (0.01 folds), IL-10 (0.40 folds), and MR (0.01 folds) was significantly reduced by LPS (1 µg/mL) + INF-γ (100 ng/mL) treatment (*p* < 0.05) while Arg-1 expression was not significantly changed as compared with that of the blank control. GOS stimulated the expression of M1 genes iNOS, IL-1β, and CCL2 in a dose-dependent manner. At low concentration levels (25 µg/mL), there were no significant effects of L-GOS on CCL-9 expression (0.71 folds). Further increment of GOS (M-GOS and H-GOS) concentration showed significantly decreased CCL-9 expression (0.21 and 0.26 folds, respectively, *p* < 0.05). GOS significantly reduced the expression of M2 genes CD163 and MR in a dose-dependent manner. Arg-1 expression was also significantly attenuated after GOS treatment while no dose-dependent manner was observed. IL-10 expression was not significantly changed by M-GOS (50 µg/mL) or L-GOS (25 µg/mL) treatment. Interestingly, H-GOS (100 µg/mL) treatment significantly increased IL-10 expression (2.37 folds, *p* < 0.05).

### 2.4. GOS Enhanced Cytokines and Nitrite Release of BMDMs

**Figure 4:**
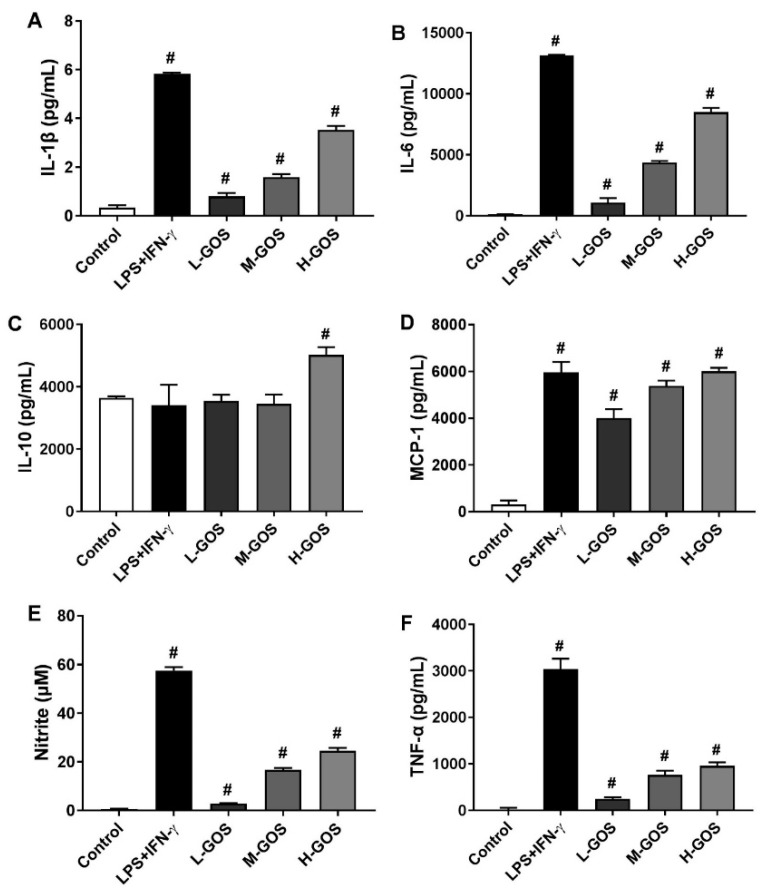
ELISA analysis of GOS (25−100 μg/mL) induced cytokine production of IL-1β (**A**), IL-6 (**B**), IL-10 (**C**), MCP-1 (**D**), and TNF-α (**F**), as well as the release of nitrite (**E**) in BMDMs. **^#^**—Indicated significant difference versus the control group at *p* < 0.05.

Effects of GOS on the secretion of cytokines [IL-1β, IL-6, IL-10, mono-cyte chemotactic protein-1 (MCP-1), and TNF-α] were investigated by enzyme-linked immunosorbent assay (ELISA), as well as the NO release of BMDMs. As shown in Figure 4, M1 macrophages were generally activated by LPS (1 µg/mL) + INF-γ (100 ng/mL) as indicated by the significantly increased release of cytokines IL-β (5.8 pg/mL), IL-6 (1.3 × 10^4^ pg/mL), MCP-1 (6.0 × 10^3^ pg/mL), and TNF-α (3.0 × 10^3^ pg/mL) as compared with those of the control (*p* < 0.05). Moreover, the NO content in the culture media of BMDMs was significantly increased after LPS (1 µg/mL) + INF-γ (100 ng/mL) treatment (57.6 µM, *p* < 0.05). However, IL-10 release of BMDMs was not significantly affected by LPS (1 µg/mL) + INF-γ (100 ng/mL) as compared with that of the control. Secretion of cytokines IL-β, IL-6, MCP-1, and TNF-α were also significantly induced by GOS in a dose-dependent manner as well as the release of NO (*p* < 0.05). However, IL-10 secretion was significantly increased by H-GOS treatment (100 µg/mL) as compared with that of the control (*p* < 0.05).

### 2.5. GOS Activated Mitogen-Activated Protein Kinases (MAPKs) and Nuclear Factor-Kappa B (NF-κB) Pathways in BMDMs

**Figure 5:**
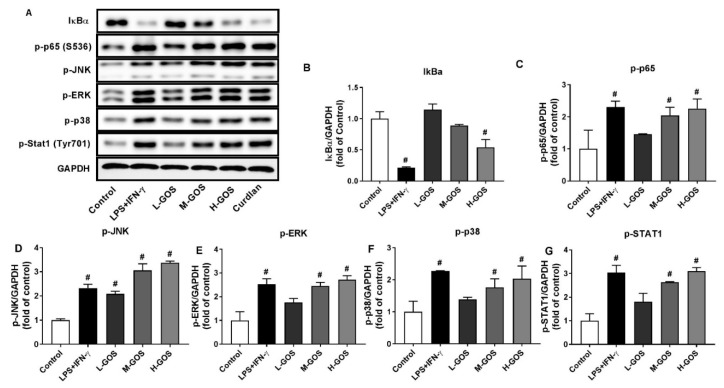
Western blotting of protein expression (**A**) and accordingly the densitometry quantification of the expressed protein bands IκBα (**B**), p-p65 (**C**), p-JNK (**D**), p-ERK (**E**), p-p38 (**F**), and p-Stat1 (**G**) in BMDMs after GOS (25−100 μg/mL) treatment. Results were expressed as the mean ± SD of three replicates. **^#^**—Indicated significant difference versus the control group at *p* < 0.05.

Molecular mechanisms underlying the polarization effects of GOS on BMDMs were investigated, including the MAPKs and NF-κB signaling pathways (Figure 5). The phosphorylation of MAPKs of c-Jun amino-terminal kinase (JNK), extracellular signal regulated kinase (ERK), and p38 in BMDMs was significantly increased after LPS (1 µg/mL) + INF-γ (100 ng/mL) treatment (*p* < 0.05). GOS increased the phosphorylation of JNK, ERK, and p38 in a dose-dependent manner as compared with that of the control (*p* < 0.05). Moreover, phosphorylation of Stat1, the downstream protein of p38 signaling pathway, was significantly increased after LPS (1 µg/mL) + INF-γ (100 ng/mL) treatment (*p* < 0.05). GOS elevated the Stat1 phosphorylation in a dose-dependent manner. LPS (1 µg/mL) + INF-γ (100 ng/mL) treatment triggered the significantly increased expression of phosphorylation of NF-κB p65 protein in BMDMs while the expression of IκBα was suppressed (*p* < 0.05). GOS increased the p65 protein phosphorylation in a dose-dependent manner (*p* < 0.05), while the IκBα expression was not significantly affected at 25 (L-GOS) and 50 µg/mL GOS (M-GOS). At 100 µg/mL, the expression of IκBα in BMDMs was significantly suppressed by H-GOS (*p* < 0.05). Activation of MAPKs and NF-κB signaling pathways could be responsible for the GOS-induced M1 polarization of BMDMs.

### 2.6. GOS Mediated M1 Phenotype Polarization was Suppressed after Receptor Blocking

**Figure 6:**
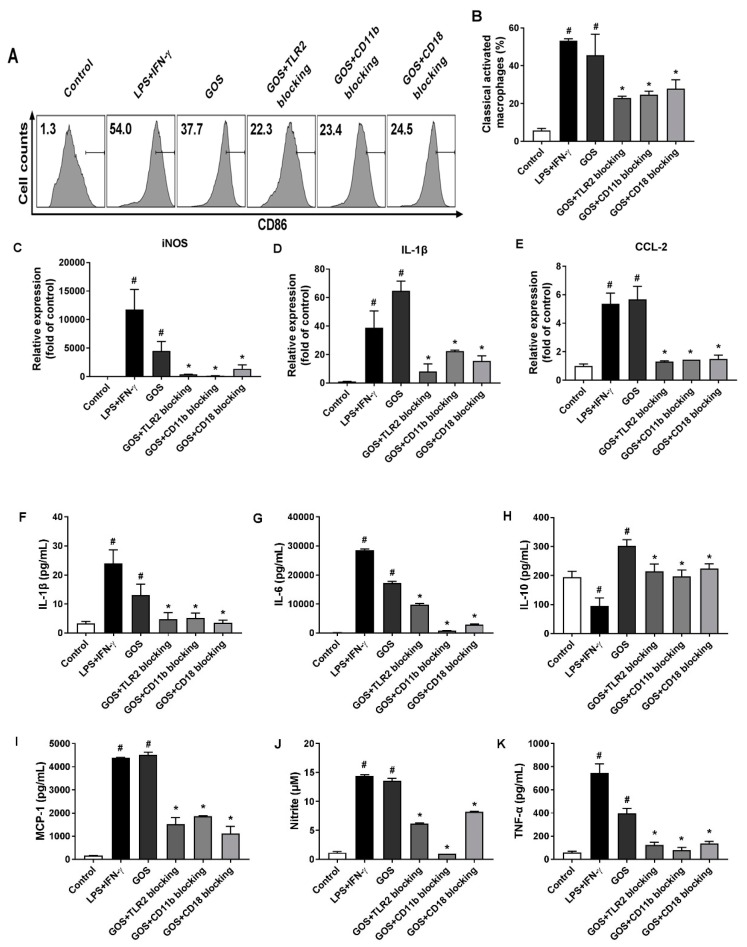
Effects of receptor blocking of TLR2 and CR3 (CD11b/CD18) on GOS-induced M1 marker expression, cytokines production, and nitrite release in BMDMs. Expression of CD86 on BMDMs cell surface (**A**) and accordingly the percentage of M1 phenotype macrophages (**B**) after receptor blocking and GOS (100 µg/mL) treatment were analyzed by flow cytometry. M1 marker gene expression of iNOS (**C**), IL-1β (**D**), and CCL-2 (**E**) were quantified by RT-qPCR. Production of cytokines of IL-1β (**F**), IL-6 (**G**), IL-10 (**H**), MCP-1 (**I**), and TNF-α (**J**), as well as the release of NO (**K**) in BMDMs were measured by ELISA kits. **^#^**—Indicated significant difference versus the control group at *p* < 0.05. *—Indicated significant difference versus the GOS group at *p* < 0.05.

To identify the receptors for GOS to induce M1 phenotype polarization, BMDMs were pretreated with anti-CR3 (CD11b/CD18) and anti-TLR2 blocking antibodies prior to GOS treatment (100 µg/mL). As shown in Figure 6, cell counts of CD86^+^ macrophages were significantly increased by LPS (1 µg/mL) + INF-γ (100 ng/mL) (54.0%, *p* < 0.05) and GOS (37.7%, *p* < 0.05) treatment, respectively, as compared with that of the control (1.3%). Pretreatment with blocking antibodies of anti-CD11b (23.4%, *p* < 0.05), anti-CD18 (24.5%, *p* < 0.05), and anti-TLR2 (22.3%, *p* < 0.05) significantly decreased the cell counts of CD86^+^ macrophages as compared with that of GOS (100 µg/mL, 37.7%). The mRNA expression of M1 genes iNOS, IL-1β, and CCL2 were significantly increased by both LPS (1 µg/mL) + INF-γ (100 ng/mL) and GOS (100 µg/mL) treatment as compared with those of the control (*p* < 0.05). However, pretreatment with anti-TLR2 and anti-CR3 (CD11b/CD18) blocking antibodies significantly attenuated the GOS (100 µg/mL) induced mRNA expression of M1 genes iNOS, IL-1β, and CCL2 (*p* < 0.05).

**Figure 7:**
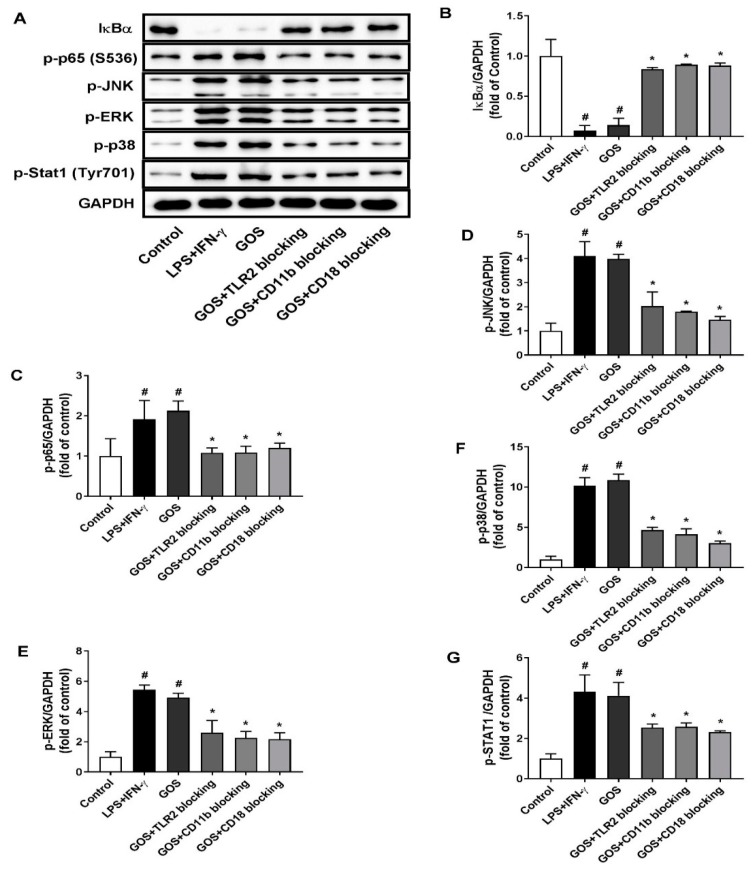
Effects of receptor blocking of TLR2 and CR3 (CD11b/CD18) on GOS activated mitogen-activated protein kinases (MAPKs) and nuclear factor-kappa B (NF-κB) signaling pathways. Western blotting of protein expression (**A**) and accordingly the densitometry quantification of the expressed protein bands IκBα (**B**), p-p65 (**C**), p-JNK (**D**), p-ERK (**E**), p-p38 (**F**), and p-Stat1 (**G**) in BMDMs pretreated with anti-CR3 (CD11b/CD18) and anti-TLR2 blocking antibodies and incubated with GOS (100 µg/mL). Results were expressed as the mean ± SD of at least three replicates. **^#^**—Indicated significant difference versus the control group at *p* < 0.05. *—Indicated significant difference versus the GOS group at *p* < 0.05.

When treated with LPS (1 µg/mL) + INF-γ (100 ng/mL) and GOS (100 µg/mL), the production of cytokines of IL-1β, IL-6, MCP-1, and TNF-α in BMDMs were significantly increased as compared with those of the control (*p* < 0.05). Meanwhile, the NO release of BMDMs was also significantly increased after both LPS (1 µg/mL) + INF-γ (100 ng/mL) and GOS (100 µg/mL) treatments (*p* < 0.05). However, the blocking antibody pretreatments with anti-CR3 (CD11b/CD18) and anti-TLR2 significantly reduced the production of cytokines IL-1β, IL-6, MCP-1, and TNF-α, as well as the NO release as compared with those of GOS (100 µg/mL, *p* < 0.05). For IL-10 production in BMDMs, LPS (1 µg/mL) + INF-γ (100 ng/mL) treatment significantly decreased IL-10 secretion (*p* < 0.05). Conversely, GOS (100 µg/mL) treatment significantly increased IL-10 production in BMDMs (*p* < 0.05). Blocking antibody pretreatments significantly decreased the IL-10 secretion as compared with that of GOS (100 µg/mL, *p* < 0.05). Accordingly, phosphorylation of MAPKs of JNK, ERK, and p38, as well as Stat1 were significantly improved by both LPS (1 µg/mL) + INF-γ (100 ng/mL) and GOS (100 µg/mL) treatments (Figure 7). Expression of NF-κB of p-p65 was also significantly increased by decreased IκBα protein expression after both LPS (1 µg/mL) + INF-γ (100 ng/mL) and GOS (100 µg/mL) treatments. Blocking antibody pretreatments significantly attenuated the expression of p-JNK, p-ERK, p-p38, p-Stat1, and p-p65, but recovered the IκBα protein expression to the control comparable level.

## 3. Discussion

Functional oligosaccharides have been claimed as prebiotics and can be prepared through extraction from natural sources, chemical or enzymatic hydrolysis of carbohydrate polymers, and organic/biological synthetic reactions [34]. The commonly recognized functional oligosaccharides include soybean oligosaccharides, fructo-oligosaccharide, isomalto-oligosaccharides, xylo-oligosaccharides, and manno-oligosaccharides [35]. They have been widely utilized as functional food components to impart beneficial health effects, particularly the immunostimulatory activity [33,34,35,36]. GOS are functional oligosaccharides that originate from curdlan. They are resistant to digestion throughout the gastrointestinal tract and show prebiotic effects to improve the growth of *Lactobacillus* strains [30]. Recently, GOS were reported to regulate the immune responses in mice treated with cyclophosphamide [32]. In the present study, we evaluated the effects of GOS on macrophage polarization and the underlying molecular mechanisms were investigated.

Depending on the surrounding microenvironmental stimuli and signals, macrophages can be polarized into pro-inflammatory M1 phenotype and anti-inflammatory M2 phenotype to mount specific functional responses of cell surface marker expression, cytokines production, and biological activities [1,2]. In this study, GOS promoted the M1 phenotype polarization of BMDMs as indicated by the upregulation of both CD11c and CD86 expression (Figure 2). This was confirmed by the increased mRNA expression of M1 genes iNOS, IL-1β, and CCL2, but suppressed mRNA expression of CD163, IL-10, and MR of BMDMs (Figure 3). Probiotic *Bacillus amyloliquefaciens* has been reported to promote M1 functional polarization of BMDMs with increased mRNA expression of M1 specific marker genes of iNOS, IL-1β, IL-6, and TNF-α [8,9]. Similar effects of the *Echinacea purpurea* extract on M1 polarization of BMDMs were observed, while the mRNA expression of M2 genes of FIZZ1, MR, Ym1, and Arg-1 were not significantly affected [12].

In general, M1 phenotype macrophages contribute to inflammation associated diseases and T-helper (Th)1-type immune response through the production of pro-inflammatory cytokines, NO, and other effector molecules [6,7]. When incubated with GOS, the secretion of IL-1β, IL-6, MCP-1, and TNF-α in BMDMs were significantly increased in a dose-dependent manner (Figure 4). In BMDMs, GOS with DP 2−4 (*Alcaligenes faecalis* var. *myxogenes*) induced production of TNF-α and IL-6. However, in DBA/2 mice, splenocytes were stimulated by GOS with increased secretion of GM-CSF and IFN-γ, but not TNF-α [31]. Paramylon derived (1→3)-β-d-glucan oligosaccharides (DP 2−7) using exo-β-1, 3-glucanases showed immunostimulatory effects with induced TNF-α production [33]. Interestingly, the production of IL-10, which typically function as an anti-inflammatory cytokine to promote the development of M2 macrophages, was significantly increased after incubation with GOS of 100 µg/mL (Figure 4). This was in accordance with the mRNA expression of IL-10 gene on BMDMs cell surface (Figure 3). However, BMDMs treated with the polysaccharide-enriched extract of *Echinacea purpurea* showed significantly increased pro-inflammatory cytokines production and accordingly the M1 gene expression. Yet there were no effects on the anti-inflammatory cytokine production of IL-10 and the M2 gene expression [12]. In the innate immune system of the host, NO could serve as the killer of infected cells, tumor cells, and parasitic pathogens [37]. Here, GOS treatment significantly increased the NO production in BMDMs. BMDMs M1 functional polarization mediated by *Bacillus amyloliquefaciens* also showed significantly increased production of NO [38].

Functionally, the secretion of pro-inflammatory cytokines by M1 macrophages can facilitate the immune responses of antigen-activated T cells against intracellular pathogens. Moreover, the upregulated superoxide bursts and NO generation increases the antimicrobial activity of M1 macrophages [3]. GOS markedly increased the pinocytic capacity and bactericidal capacity of BMDMs without cytotoxicity (Figure 1). Similar results were reported for glycyrrhizic acid derived from the roots and rhizomes of *Glycyrrhiza* species (licorice) which induce the M1 macrophage polarization of BMDMs with enhanced uptake of FITC-dextran and *Escherichia coli* K88 [39]. Thus, GOS may have protective effects against intracellular infections through the M1 functional polarization of macrophages.

MAPKs play a vital role in macrophage activation and differentiation. The phosphorylation of transcription factors of ERK, JNK, and p38 MAPKs are a prerequisite for the cellular responses to environmental stimuli, such as NO release and production of various pro-inflammatory cytokines [40,41]. GOS treatment induced the phosphorylation of all three MAPKs of ERK, JNK, and p38 in BMDMs (Figure 5). Subsequently, the activation of NF-κB, a pivotal transcription factor in immune and inflammation mediation, was regulated by MAPKs with the detachment of ΙκB protein and phosphorylation of p65 protein. The free NF-κB would further trigger the gene expression of cytokines through the translocation from cytoplasm to nucleus, and thus the immune responses [42]. The expression of IκBα protein in BMDMs was significantly suppressed by GOS treatment while the phosphorylation of p65 protein was remarkably increased (Figure 5). Together, GOS triggered the activation of both MAPK and NF-κB signaling pathways and induced the M1 polarization of BMDMs with increased release of NO and pro-inflammatory cytokines. *Astragalus* polysaccharide (RAP) was also found to induce the M1 polarization of RAW264.7 cells through the activation of both MAPK and NF-κB signaling pathways. Moreover, the notch signaling pathway proved to be involved in the RAP-induced macrophage M1 polarization [16]. However, the polysaccharide-enriched extract of *Echinacea purpurea* polarized BMDMs into M1 phenotype only through JNK signaling pathway [12].

Identification of the receptors for GOS is critically important for the elucidation of molecular mechanisms underlying the induced M1 phenotype polarization of BMDMs. Usually, a broad range of receptors are expressed on the macrophage cell surface, such as TLRs and CR3 (CD11b/CD18) [42]. They can interact with immune stimulants and induce the activation of the downstream MAPKs and NF-κB signaling pathways, thus leading to the release of inflammatory cytokines and NO [43]. Therefore, effects of receptor blocking with antibodies of anti-TLR2 and anti-CR3 (CD11b/CD18) on GOS-induced BMDMs polarization were examined. After receptor blocking, GOS-induced M1 phenotype polarization of BMDMs was diminished (Figure 6). Accordingly, the mRNA expression of M1 marker genes iNOS, IL-1β, and CCL-2 was suppressed with the reduced release of cytokines IL-1β, IL-6, MCP-1, and TNF-α, as well as NO (Figure 6). Those could be ascribed to the suppressed activation of MAPKs and NF-κB as indicated by the attenuated phosphorylation of transcription factors (Figure 7). Thus, TLR2 and CR3 (CD11b/CD18) on BMDMs could both recognize GOS and active the immune responses. However, for the RAW264.7 cells, GOS have been reported to interact with the receptors of TLR2 and CD18 to activate the macrophages, but independent with that of CD11b [32], while both CR3 (CD11b/CD18) and TLR2 were responsible for the recognition of linear β-glucan from the baker’s yeast to activate RAW264.7 cells [44].

## 4. Materials and Methods

### 4.1. Materials

Curdlan from *Alcaligenes faecalis* (food grade) with DP of 400−500 was provided by Jiangsu Yiming Biological Technology Co., Ltd. (Taixing, Jiangsu, China). GOS (DP = 2, 26.39%; DP = 3, 40.65%; DP = 4, 23.78%; DP = 5, 9.18%) were produced through enzymatic hydrolysis using GH family 64 β-(1→3)-(*Rm*Lam81A) [30,45]. RPMI 1640 medium was obtained from Corning (Manassas, VA, USA). NO assay kit was obtained from Beyotime Biotech. (Haimen, Jiangsu, China). Assay kits for TNF-α, IL-1β, IL-10, MCP-1, IL-6, anti-CD11b antibody, anti-CD18 antibody, anti-TLR2 antibody, anti-TLR4 antibody, CD16/32, Brilliant violet 421 labeled mouse anti-CD11c (N418), and PE/Cy7 labeled mouse anti-CD86 antibody were obtained from Biolegend (San Diego, CA, USA). Antibodies against p-p65 (S536), IκBα, p-JNK, p-ERK, p-p38, and Stat1 (S727) were purchased from Cell Signaling Technology (Beverly, MA, USA). Macrophage colony-stimulating factor, LPS, and fluorescein isothiocyanate (FITC)-dextran (average molecular weight of 40,000) were received from Sigma-Aldrich (St. Louis, MO, USA). IFN-γ was purchased from Peprotech (Rocky Hill, NJ, USA). All other chemicals were used as received without further purification.

### 4.2. Isolation and Differentiation of Bone Marrow Cells

Healthy male, 7-week-old C57BL/6J mice were obtained from Vital River Laboratories (Beijing, China) and raised in polypropylene cages under standard laboratory conditions (light/dark cycle of 12 h/12 h, temperature of 25 ± 2 °C, and relative humidity of 55 ± 5%). Standard diet (16% protein, 60% carbohydrates, 3% fat, 5% minerals and ash, and 12% moisture) and water were supplied ad libitum. The animal handling protocol was approved by the Institutional Animal Ethics Committee of China Agricultural University.

After one week adaptation, mice were sacrificed, and bone marrow cells were isolated from aspirates of tibiae and femurs. The cells were flushed with sterilized phosphate buffered saline (PBS, pH 7.4) and the red blood cells were lysed. After centrifugation and filtration through a cell strainer, cells were resuspended and incubated in a 100 mm cell culture plate containing RPMI-1640 media. Subsequently, BMDMs differentiation was induced by incubation with macrophage colony-stimulating factor (100 ng/mL) (Peprotech, Rocky Hill, NJ, USA) in a humidified atmosphere (5% CO_2_ and 95% humidity) at 37 °C for six days. BMDMs were then harvested and seeded for subsequent analyses.

### 4.3. Cytotoxicity Assay

BMDMs were seeded in 96-well plates at a density of 1 × 10^5^ cells/well and incubated with GOS (5–1000 μg/mL) and curdlan (5–1000 μg/mL) for 24 h in a cell incubator (5% CO_2_, 95% humidity, and 37 °C). Supernatants of the cell suspension were replaced with RPMI-1640 media containing 0.5 mg/mL MTT. After incubation for 4 h, the supernatants were removed, and the precipitates were dissolved in DMSO. Optical density at 570 nm (OD_570_) was measured using a microplate reader for relative cell viability quantification.

### 4.4. Pinocytosis and Bacterial Killing Assays

Pinocytosis of BMDMs was measured as the cellular uptake of FITC-dextran using flow cytometry (CyAn flow cytometer, Beckman Coulter, Atlanta, GA, USA). BMDMs (1 × 10^5^ cells/well in 96-well plate) were incubated with LPS (1 µg/mL) plus IFN-γ (100 ng/mL) (LPS+IFNγ), 25 µg/mL GOS (L-GOS), 50 µg/mL GOS (M-GOS), and 100 µg/mL GOS (H-GOS), respectively, for 12 h at 37 °C. Cells incubated with PBS were treated as the blank control. Then, cells were exposed to FITC-dextran (1 mg/mL) for 1 h and washed three times with PBS to remove the excess dextran. The percentage and mean fluorescence intensity (MFI) of intracellular FITC-dextran were measured for pinocytic index (PI) calculation [46].

To study the effects of GOS on bactericidal function of BMDMs, cells were seeded in 24-well culture plates (2 × 10^5^ cells/well) and incubated with GOS for 24 h. Then, BMDMs were incubated with 2 × 10^7^ *S. typhimurium* (strain CMCC-50115) for 1 h at 37 °C. The extracellular bacteria were then killed by gentamicin (25 µg/mL) in Dulbecco′s modified Eagle medium (24 h) and thoroughly washed with PBS. Finally, BMDMs were lysed with Triton X–100 in PBS (1%, *w*/*v*) and the lysates were serially diluted with PBS and plated onto Luria broth (LB) agar in triplicate. The CFUs of intracellular bacteria was counted to determine the bactericidal capacity [39].

### 4.5. Flow Cytometric Analysis

Surface expression of CD11c and CD86 on BMDMs were measured to examine the cell phenotypes. BMDM cells (1 × 10^5^ cells per well in a 96-well plate) were collected by pipetting in PBS with 1 mM EDTA after sample treatment (LPS + IFNγ, L-GOS, M-GOS, and H-GOS) and washed twice with cold PBS. The collected cells were then blocked with CD16/32 antibody and stained with Brilliant violet 421 labeled mouse anti-CD11c (N418) antibody and PE/Cy7 labeled mouse anti-CD86 antibody for 30 min at 4 °C, respectively. After staining, the cells were immediately fixed with formaldehyde (2%, *w*/*v*) and the surface expression of CD11c and CD86 was analyzed on a CyAn flow cytometer (Beckman Coulter) using FlowJo V10 software (Atlanta, GA, USA).

### 4.6. Nitrite and Cytokine Production Measurement

After each treatment, supernatants of BMDMs cell culture were collected. The nitrite accumulation in the cell culture supernatant was measured by the Griess method [47]. Production of inflammatory cytokines of TNF-α, IL-1β, IL-10, MCP-1, and IL-6 in the collected supernatants were determined by ELISA kits (Biolegend, San Diego, CA, USA). All analyses were performed following the manufacturer’s instructions.

### 4.7. Quantitative Real Time-Polymerase Chain Reaction

Extraction of total RNA from BMDMs after each sample treatment (LPS + IFNγ, L-GOS, M-GOS, and H-GOS) was conducted using TRIzol reagent (Thermo Fisher Scientific, Waltham, MA, USA) according to the manufacturer’s protocol. Then, 1 μg of total RNA was subjected for cDNA generation through reverse transcription reaction using a GoScript™ Reverse Tracription Kit (Promega, Madison, WI, USA). The mRNA expression level was measured using the TB Green™ Premix Ex Taq™ II (Tli RNaseH Plus, TAKALA) and CFX96 (Bio-Rad, Hercules, CA, USA) with the generated cDNA as the template. ARG-1, IL-10, CD163, CD206, IL-1β, iNOS, CCL-2, CCL-9, and GAPDH were amplified through the LightCycler^®^ 96 real RT-qPCR system (Roche, Mannheim, Germany). The conditions of the RT-qPCR cycle were as follows: initial hold step, 30 s at 95 °C, followed by 40 cycles of 5 s at 95 °C, 60 °C for 30 s, and 72 °C for 30 s. Melting curves were obtained stepwise from 55 °C to 95 °C. The fold induction of each targeted gene expression was calculated through the comparative method (2^−ΔΔCt^), following normalization with the internal control GAPDH. The primers for the target genes are listed in Table 1.

### 4.8. Western Blot Assay

Protein expression of IκBα and phosphorylation of p65 (S536), JNK, ERK, p38, and Stat1 (Tyr701) was analyzed by Western blotting. BMDMs were lysed using radio immunoprecipitation assay buffer (100 μL) supplemented with a 1/200 dilution of protease and phosphatase inhibitor cocktails. The cellular lysates were centrifuged at 12,000× *g* and 4 °C for 15 min after 30 min of lysis on ice. The protein content in the supernatant was measured by bicinchoninic acid (BCA) method [38]. Fifty micrograms of protein were loaded onto 12% sodium dodecyl sulfate (SDS)-polyacrylamide gel for electrophoresis, and then electrophoretically transferred onto nitrocellulose membranes (Cell Signaling Technology Inc., Danvers, MA, USA). The membranes were subsequently blocked with nonfat milk (5% in Tris-buffered saline containing 0.1% Tween 20, TBS-T) for 1 h at 37 °C. The pre-blocked membranes were incubated with specific primary antibodies of p-p65 (S536), IκBα, p-JNK, p-ERK, p-p38, Stat1 (Tyr701), and GAPDH at 4 °C overnight. Immunoblots were washed three times with TBS-T (each for 5 min) and incubated with horseradish peroxidase (HRP)-conjugated goat anti-rabbit immunoglobulin IgG (Cell Signaling) at room temperature for 1 h. The protein bands were visualized and analyzed using ChemiDoc XRS system (Bio-Rad).

### 4.9. Receptor Blocking Assay

Assays of receptor blocking were performed as previously described [44]. BMDMs were cultured in 96-well plates at a cell density of 2 × 10^6^ cells/well. The cells were then treated with monoclonal antibodies against anti-CD11b (5 μg/mL), anti-CD18 (5 μg/mL), and anti-TLR2 (5 μg/mL) at 4 °C for 30 min. Subsequently, GOS (100 μg/mL) were added and cells were incubated for another 24 h at room temperature. Cells were collected for ELISA, qRT-PCR, and Western blot assays as described above.

### 4.10. Statistical Analysis

All experiments were performed in triplicate. Data were presented as the mean ± standard deviation (SD). Statistical differences were tested by one-way analysis of variance (ANOVA) and Tukey’s test. Probability value of *p* < 0.05 was considered as significant.

## 5. Conclusions

Molecular mechanisms involved in the GOS induced macrophage polarization were studied. GOS were non-toxic to BMDMs and induced the M1 phenotype polarization with increased pinocytic capacity and bactericidal capacity, mRNA expression of M1 marker genes, release of proinflammatory cytokines, and nitrite. BMDMs polarization was mediated through MAPKs and NF-κB signaling pathways activation induced by GOS. Furthermore, receptors of CR3 (CD11b/CD18) and TLR2 were responsible for the recognition of GOS and downstream signaling pathway activation. Findings gained in this study would expand the utilization of GOS as a natural immunomodulator in functional foods.

## Figures and Tables

**Table 1:** Primer sequences for real time-quantitative polymerase chain reaction (RT-qPCR).

Target Genes	Primer sequences (5′−3′)	
	Forward	Reverse
ARG-1	TGAGAGACCACGGGGACCTG	GCACCACACTGACTCTTCCATTC
IL-10	CTCTTACTGACTGGCATGAGGAT	GAGTCGGTTAGCAGTATGTTGT
CD163	CCTCCTCATTGTCTTCCTCCTGTG	CATCCGCCTTTGAATCCATCTCTTG
CD206	TCAGCTATTGGACGCGAGGCA	TCCGGGTTGCAAGTTGCCGT
IL-1β	GCAACTGTTCCTGAACTCAACT	ATCTTTTGGGGTCCGTCAACT
iNOS	CACCTTGGAGTTCACCCAGT	ACCACTCGTACTTGGGATGC
CCL-2	TTAAAAACCTGGATCGGAACCAA	GCATTAGCTTCAGATTTACGGGT
CCL-9	CCCTCTCCTTCCTCATTCTTACA	AGTCTTGAAAGCCCATGTGAAA
GAPDH	GTGCTGAGTATGTCGTGGAGTCTAC	GGCGGAGATGATGACCCTTTTGG
